# An unusual appearance of the post-pubertal Herlyn-Werner-Wunderlich syndrome with acute abdominal pain: A case report

**DOI:** 10.18502/ijrm.v17i10.5498

**Published:** 2019-11-28

**Authors:** Marzieh Ghasemi, Arezoo Esmailzadeh

**Affiliations:** ^1^Department of Obstetrics and Gynecology, Pregnancy Health Research Center, Zahedan University of Medical Sciences, Zahedan, Iran.; ^2^Department of Obstetrics and Gynecology, Trauma Research Center, Baqiyatallah University of Medical Sciences, Tehran, Iran.

**Keywords:** Herlyn-Werner-Wunderlich syndrome, Uterus didelphys, Kidney agenesis, Mullerian duct anomaly.

## Abstract

**Background:**

Herlyn-Werner-Wunderlich (HWW) syndrome is a rare congenital urogenital defect. It is detected by unilateral low vaginal obstruction, uterus didelphys, and ipsilateral kidney agenesis. It usually becomes apparent with pain, dysmenorrhea, and presence of a vaginal or pelvic mass. Purulent vaginal discharge may also happen rarely because of infective complications of the obstructed hemivagina. In this report, we describe a post-pubertal case with acute abdominal pain.

**Case:**

The patient was a 13-yr-old girl who was referred to us with acute abdominal pain one year after the onset of her menarche. In the pelvic examination, we detected hematocolpos. Abdominopelvic-computed tomography scan confirmed the presence of mullerian duct anomalies with uterus didelphys. This case of HWW syndrome along with pyocolpus was managed by vaginal septum resection, drainage of pus, and salpingectomy.

**Conclusion:**

The symptoms of HWW syndrome should be monitored in early puberty to prevent more complications.

## 1. Introduction

Herlyn-Werner-Wunderlich (HWW) syndrome is a rare congenital obstructive mullerian anomaly with 0.1-3.8% prevalence in women. It is detected by hemilateral vaginal obstruction, uterus didelphys, and ipsilateral kidney agenesis (1, 2). It is caused by lateral non-fusion of the mullerian ducts in the one-sided obstructed vagina (2, 3). Usually patients are asymptomatic before puberty; however, the symptoms become present gradually because of the acute congestion of blood in the vagina after menarche (4). This syndrome is characterized by a triad of uterus didelphys, ipsilateral kidney agenesis, vaginal septum, and hemilateral vaginal obstruction (5). Uterus didelphys is class 3 anomaly of the American Fertility Society classification (5). Most patients are adolescent girls with pelvic pain and mass after menarche (1). In a few cases, pyohematocolpos, pyosalpinx, and peritonitis may appear because of the ascending infection in the obstructed hemivagina (6). In the pelvic examination, hematocolpos is seen with a visible bulge in the vagina (6). Although the ultrasound imaging is the first choice for detection, magnetic resonance imaging is suggested for complementary and definitive diagnosis (4). Laparotomy and laparoscopy are used in acute cases only (1).

In this report, we described an unusual case of a young girl with HWW syndrome with pyocolpus, peritonitis, and endometriosis that was successfully treated by resectioning vaginal septum, salpingectomy, and pus drainage.

## 2. Case Presentation

Our patient was a 13-yr-old girl who was referred to our hospital because of acute abdominal pain. She had a fever for two weeks before being referred to us. Her menarche had begun one year before her referral. She had suffered from severe dysmenorrhea until then, and her last menstrual cycle had been two days before coming to the hospital. Her abdominal pain had increased during her last menstruation. The patient had a fever, chills, abdominal pain, and nausea at the time of admission to the hospital. She did not have a vaginal discharge with bad odor and had no particular problems in her medical history. Generalized abdominal tenderness was diagnosed in the physical examination, but mild vaginal bleeding and right lateral vaginal wall bulging were found in her vaginal and transrectal examinations. She underwent laboratory tests including leukocytosis (18,000 mm), platelet (441,000), hemoglobin (10.7), C-reactive protein (3+), normal liver and kidney function tests, and normal coagulate profile tests. Her abdominal ultrasound images showed uterus didelphys with a mixed-pattern cystic mass measuring 72 × 65 × 62 mm close to the right ovary plus a solid cystic mass measuring 100 × 58 mm in the right adnexa. The right kidney was not observed.

We decided to do abdominal and pelvic computed tomography scan to evaluate the cystic mass which was reported in abdominal ultrasound imaging. We had planned to do the surgery in an appropriate condition. This meant with patient's intestinal preparation after evaluating computed tomography results, and in the presence of an expert general surgeon. However, a few hours after the admission, we had to do an emergency surgery on the same night because she had a high fever, increased ESR and CRP, and a possible risk of septic shock. We administered many antibiotics before the emergency surgery because of the acute abdominal pain.

During the first surgery, we did a physical examination while she was under anesthesia. The hymen was normal and intact. But a buldge was found in the right side of her vagina during the rectoabdominal examination. This was confirmed by vaginal examination with a speculum. The right longitudinal septum was revealed with a closed end. At first, we cut the closed end of the hemilateral obstruction of the vagina and then the vaginal septum was resected with an electrocautery device. Afterward, a 200-cc pus was drained out, and this sample was sent to a laboratory for culture and antibiogram.

The patient's abdomen was opened with a midline incision. There was a severe adhesion of the intestine and omentum to the abdominal wall. Since no intestinal preparation had been done and there was a high risk of intestinal rupture, the general surgeon decided that it would be better to end this surgery and prepare the patient for another abdominal surgery in better conditions.

**Figure 1:**
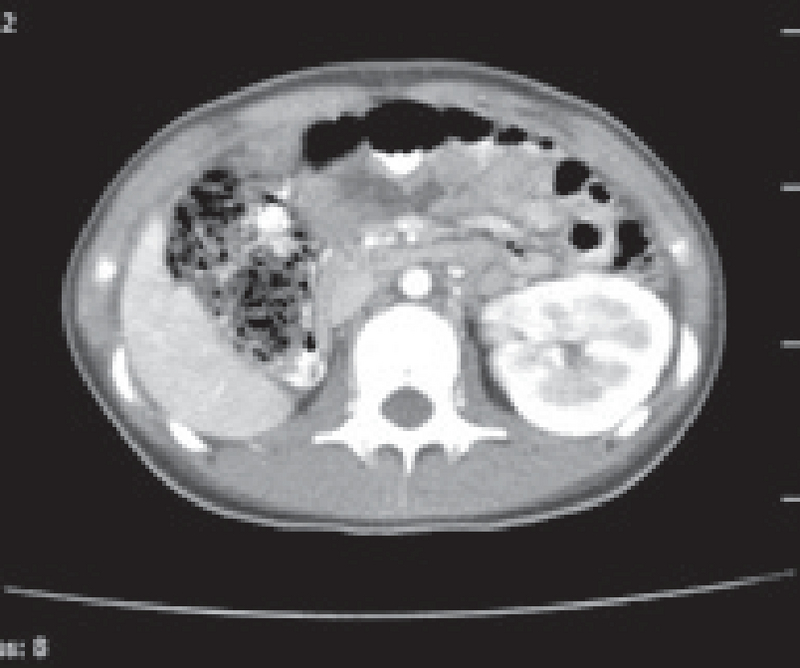
Abdominopelvic CT-scan: multi-cystic mass with severe development in the right adnexa.

**Figure 2:**
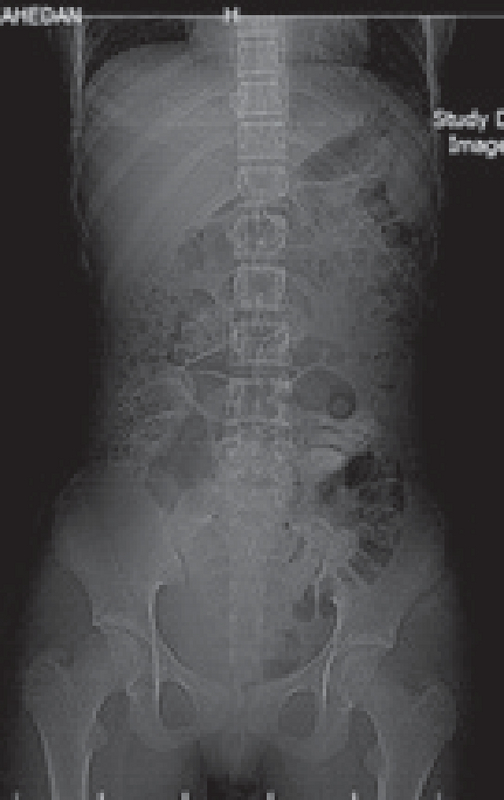
Abdominopelvic CT-scan.

Abdominopelvic-computed tomography scan (with and without contrast which was done after the first surgery) confirmed the ultrasound imaging findings of uterus didelphys with multi-cystic mass with severe development in the right adnexa. A remarkable right hematocolpus hematosalpynx was seen that was due to longitudinal vaginal septum and hemivaginal obstruction. The right kidney was not observed (Figures 1, 2). Her problem was detected as HWW syndrome infected with pyocolpos.

We did the second laparotomy in less than 48 hr after the first surgery with the same incision in the presence of an expert surgeon. Our patient had severe intestinal adhesion and perforated appendix fistulated to the right fallopian tube beside distal small intestinal obstruction. We saw pelvic inflammatory mass beside endometriosis. We did an appendectomy, right salpingectomy, entrolysis, and abscess drainage. Cefepim (Maxipim) and cloxacillin (Jaber-ibn-Hayyan pharmaceutical co) were administrated intravenously as specific infectious disease drugs until seven days after the surgery according to the specimen culture. We ordered continuous low-dosage contraceptive drugs for preventing the progression of endometriosis. Our patient was discharged from the hospital after seven days with a good condition.

## 3. Ethical Consideration

After the completion of treatment, we asked the patient for the permission to publish her anomaly as a case report. She accepted and gave us an oral consent.

## 4. Discussion

The female genital system has two mullerian ducts fused in the midline with urogenital sinus (6). Failure of resorption during this fusion leads to an obstructive anomaly of mullerian system (4, 6). A rare example of this obstructive anomaly is the HWW syndrome (3, 7). This syndrome was explained by Herlyn-Werner in 1971 for the first time and it was completed by Wunderlich in 1976 (8). In this syndrome, obstructed hemivagina is the main reason for blood accumulation in the uterus and retrograde bleeding in the abdomen through fallopian tubes that can cause pelvic pain and mass, hematometra, hematocolpus, and finally endometriosis. The symptoms at the time of being referred to the hospital depends on the degree of the anomaly. It consists of dysmenorrhea, urinary incontinence, infertility, and acute abdominal pain due to endometriosis (4, 6, 9).

Dongan and colleagues classified the HWW syndrome into two types: complete or incomplete vaginal septum (4). Tong and colleagues stated that the age of pelvic endometriosis onset is earlier than the age of finding endometriosis. Approximately, in 88.89% of HWW syndrome cases, the patients are affected by endometriosis (9). They are usually diagnosed in the pubertal or post-pubertal period, but Angotti and colleagues reported six cases of this problem in girls younger than five years old (8). The resectioning of the vaginal septum in obstructive hemivagina should be done through an abdominal or transvaginal approach (8).

In our case after confirming the HWW syndrome with the computed tomographic scan, we did a vaginal septum resection. Our case was complicated because of pyosalpinx. So, investigative laparotomy was done. A two-step surgery is recommended in similar cases by pyocolpus for a complete vaginal septum resection (6). Sometimes, the closure of an opening will occur at the end of one year (4). Doing a complete vaginal septum resection is necessary for assuring the future fertility, while uterus didelphys has no effect on reducing the infertility. So, fertility prognoses remain good after surgery in uncomplicated cases (2, 4).

This report described a case of the unusual type of HWW syndrome with fever and acute abdominal pain. The main complaint of our patient was only severe dysmenorrhea. Cyclic abdominal pain, abdominal mass, dysmenorrhea, with or without urinary disorders, can lead to this problem in adolescence. So, the symptoms should be emphasized in early puberty to prevent more complications. Well-timed removal of the vaginal septum can improve fertility and endometriosis. Also, it decreases pyosalpinx, acute abdominal pain, and further surgeries.

A limitation of our work was doing the surgery twice. If the patient was in a better condition, we could have done the surgery same as many other cases with opening the obstruction of vagina and laparoscopy. However, because of the different situation of the studied case and that she had a acute abdominal pain that could be a sign of infection, we tried to avoid sepsis with doing the surgery in two steps.

## 5. Conclusion

Although dysmenorrhea is among girls in puberty, it is advised to follow-up acute dysmenorrhea soon to avoid further medical consequences.

##  Conflict of Interest

The authors report no conflicts of interest.

## References

[1] Ma I., Williamson A., Rowe D., Ritchey M., Graziano K. (2014). OHVIRA with a twist: obstructed hemivagina ipsilateral renal anomaly with urogenital sinus in 2 patients. *J Pediatr Adolesc Gynecol*.

[2] Sanghvi Y., Shastri P., Mane S., Dhende N. P. (2011). Prepubertal presentation of Herlyn-Werner-Wunderlich syndrome: a case report. *Journal of Pediatric Surgery*.

[3] Güdücü N., Gönenç G., İşçi H., Yiğiter A. B., Dünder İ. (2012). Herlyn-Werner-Wunderlich Syndrome—Timely Diagnosis is Important to Preserve Fertility. *Journal of Pediatric & Adolescent Gynecology*.

[4] Dogan A., Uyar I., Demirtas G. S., Ekin A., Gulhan I., Ertas I. E., Ozeren M. (2016). Urinary Incontinence in Puberty: A Rare Clinical Presentation of the Herlyn-Werner-Wunderlich Syndrome. *Journal of Pediatric & Adolescent Gynecology*.

[5] Yavuz A., Bora A., Kurdoğlu M., Goya C., Kurdoğlu Z., Beyazal M., Akdemir Z. (2015). Herlyn-Werner-Wunderlich Syndrome: Merits of Sonographic and Magnetic Resonance Imaging for Accurate Diagnosis and Patient Management in 13 Cases. *Journal of Pediatric & Adolescent Gynecology*.

[6] Jung E. J., Cho M. H., Kim D. H., Byun J. M., Kim Y. N., Jeong D. H., Sung M. S., Kim K. T., Lee K. B. (2017). Herlyn-Werner-Wunderlich syndrome: An unusual presentation with pyocolpos. *Obstetrics & Gynecology Science*.

[7] Yung S. S., Ngu S., Cheung V. Y. (2016). Late presentation of a variant of Herlyn-Werner-Wunderlich syndrome. *International Journal of Gynecology & Obstetrics*.

[8] Angotti R., Molinaro F., Bulotta A. L., Bindi E., Cerchia E., Sica M., Messina M. (2015). Herlyn-Werner-Wunderlich syndrome: An "early" onset case report and review of Literature. *International Journal of Surgery Case Reports*.

[9] Tong J., Zhu L., Chen N., Lang J. (2014). Endometriosis in association with Herlyn-Werner-Wunderlich syndrome. *Fertility and Sterility*.

